# Racial disparities in the development of comorbid conditions after preterm birth: A narrative review

**DOI:** 10.1016/j.semperi.2022.151657

**Published:** 2022-08-28

**Authors:** Kayla L. Karvonen, Faith Goronga, Safyer McKenzie-Sampson, Elizabeth E. Rogers

**Affiliations:** a Department of Pediatrics, University of California San Francisco, San Francisco, CA, United States; b California Preterm Birth Initiative, University of California San Francisco, San Francisco, CA, United States; c Department of Epidemiology and Biostatistics, University of California San Francisco, San Francisco, CA, United States

## Abstract

Despite recognition and attempts to reduce racial disparities in perinatal outcomes, Black infants are still disproportionately represented among those who are born preterm. Postnatal investigations of racial disparities in comorbidities and outcomes after preterm birth are increasing, although their results and interpretations are conflicting. In the present review, we 1.) identify important methodological limitations of that literature 2.) summarize the conflicting literature investigating racial disparities, specifically Black-white differences, in postnatal comorbidities and outcomes after preterm birth 3.) describe mechanisms by which racism operates to contextualize our understanding to inform future work to actively reduce disparities in preterm birth and subsequently, its complications.

## Background

Racial and ethnic disparities in perinatal outcomes including infant mortality, preterm birth, and low birthweight have long been described, with Black families persistently at highest risk of each.^1,2^ Unequal distributions of social determinants of health and exposure to stress, racism, and discrimination are known risk factors for poor perinatal outcomes for Black birthing people.^3–7^ Historically, researchers proposed that genetic differences accounted for disparities in perinatal outcomes, a theory that has since been debunked. Race is a social construct without a genetic basis, and thus genetics cannot be understood as a root cause of racial disparities. Instead, racism in all of its forms is increasingly being recognized as a significant driver of racial disparities.^8,9^ Despite recognition and attempts to reduce racial disparities in perinatal outcomes, inequities have persisted, resulting in a disproportionate proportion of Black infants who are born preterm.^10,11^ Postnatal investigations of racial disparities in comorbidities and outcomes after preterm birth are increasing, although their results and interpretations are conflicting.

Technological advancements like antenatal steroids, surfactant administration, mechanical ventilation, and continuous positive airway pressure machines (CPAP) have conferred increased survival for preterm infants over time.^12,13^ Despite advancements in the field of neonatology, preterm infants, especially those born extremely preterm, are at significant risk of comorbid conditions such as respiratory distress syndrome (RDS), bronchopulmonary dysplasia (BPD), persistent pulmonary hypertension (PPHN), necrotizing enterocolitis (NEC), intraventricular hemorrhage (IVH), retinopathy of prematurity (ROP), patent ductus arteriosus (PDA), and sepsis. Preterm comorbidities have a wide range of potential impact on preterm infants and their families in regards to neurodevelopment, disability, and finances. For some families, the effect of preterm comorbidities can be severe with lasting consequences into childhood and adulthood.^14–20^

For many years, there has been a belief in the neonatal community that Black preterm infants have a morbidity and mortality advantage over their white counterparts.^21–23^ In the present review, we 1.)identify important methodological limitations of prior literature 2.) summarize conflicting literature investigating racial disparities in comorbidities and outcomes after preterm birth, specifically focusing on Black-white disparities (Supplemental Table 1) 3.)describe mechanisms by which racism operates to contextualize our understanding and to inform future work to actively reduce disparities in preterm birth and subsequently, its complications.

## Methodologic considerations

There is considerable variability in the published literature regarding racial disparities in comorbidities associated with preterm birth, and it is important to note the methodological limitations and variations of past studies as it relates to conflicting interpretations of results.

As an example, the literature is inconsistent regarding Black-white disparities found in NEC. Some studies have found that there is an increased risk of NEC among Black infants compared to white infants, where others have found no relationship between race and NEC.^24,25^ Differences between racial groups in incidence of NEC is physiologically plausible, given racial differences in major risk factors for NEC such as provision of breast milk, antenatal steroids, and fetal growth, yet studies remain conflicting.^26–33^

It has been suggested that these opposing findings may be due to differences in statistical methodology.^24,34^ Historically, cohort studies of racial disparities in preterm comorbidities have employed conventional logistic regression analyses to yield their effect estimates.^11,25,35–41^ These cohort studies typically stratified by GA to select the study sample, therefore incurring collider stratification bias.^24^ Collider stratification bias occurs when the association between the exposure and outcome is induced by stratification on an intermediate variable, which is a common cause of the exposure and outcome. ^42^ A classic example of collider stratification bias in studies of perinatal outcomes is the “birth weight paradox”, wherein it appears that maternal smoking has a protective effect on mortality for low birthweight (LBW) infants. However, this paradoxical effect appears due to invalid stratification by birth weight, which induces a spurious association between maternal smoking and mortality.^42,43^ This occurs because the consequences of being <2500 g from maternal smoking are less dire than when the cause is preterm birth or a genetic syndrome.

One strategy to reduce collider stratification bias is the fetus-at-risk approach. The fetus-at-risk approach includes in the denominator for any given outcome at a given gestational age (GA) not only the delivered infants, but also ongoing pregnancies.^44–47^ Moreover, the fetus-at-risk approach is preferable when the risk factor (in this case, socially-assigned race) occurs before birth. Janevic et al. demonstrated that by applying the fetus-at-risk approach to their cohort, racial disparities were identified that were previously underrecognized.^24^ Black infants were at differentially higher risk of various preterm comorbidities including NEC, IVH, BPD, and ROP, which was previously unrecognized using a conventional analytic approach.^24,34^ Nevertheless, the use of a fetus-at-risk approach should be ultimately motivated by the research question and the perinatal outcomes of interest, as this method may not always be appropriate.^48^ For example, the use of fetus-at-risk approach once a preterm infant is born is no longer valid to counsel parents about the likelihood of developing postnatal comorbidities.

We also found that several studies defined their exposure of interest, infant race, as being the same race of the birthing parent.^25,40^ This is concerning as researchers have found this method may bias the assessment of the outcomes under investigation.^49–51^ The potential for exposure misclassification is particularly amplified in cases where the infant is multiracial, and it is recommended that future work critically analyze and acknowledge the limitations of infant race obtained from administrative databases.^51^

Finally, to add to the complexity of interpretation, the studies included in this narrative review vary in their patient populations, inclusion criteria, study design, outcome definitions, time period of study, and sample sizes, which in turn limits the statistical power to detect meaningful differences and may contribute to the dissimilar findings (Table 1).

## Infant mortality

Before reviewing preterm comorbidities, we will briefly summarize infant mortality, as mortality and morbidity are competing outcomes. For example, the preterm infant who dies early in life will not go on to develop BPD. A more thorough summary can be found in this issue by Driscoll et al.^149^ Black birthing people experience more fetal and perinatal loss compared to other racial and ethnic groups.^49^ Periviable births contribute significantly to infant mortality, and Black birthing people are 3–6 times more likely to experience periviable fetal loss compared to white birthing people.^52^ In the periviable period, a survival advantage for Black infants was historically described in the 1990s, that not only diminished but transitioned to a widening survival disadvantage for Black infants.^53,54^ Preterm-related infant mortality was three times higher for Black infants compared to white infants from 2005 to 2013, and although preterm-related infant mortality is on the decline overall, the rate of decline is slower for Black infants compared to white infants.^1,2^ Regarding in-hospital mortality, several studies have not described a survival disadvantage for Black infants.^11,25,55,56^ While infant mortality is certainly a significant outcome, comorbidities of preterm birth have the potential for impactful long-term individual and societal consequences as they can evolve into adverse neurodevelopment, chronic illness, and emotional and financial stress over the life course.

## Preterm co-morbidities

### Respiratory Distress Syndrome

Surfactant deficiency is a result of immature surfactant synthesis and function in the premature lung and is the primary cause of RDS observed in preterm neonates. RDS can result in progressive atelectasis and respiratory failure without intervention and is a common comorbidity of preterm birth, with incidence ranging from 85–95% for 24–28-week GA infants.^57^ Most studies have shown either no difference or lower rates of RDS among Black neonates compared to white neonates including a large cohort study of infants using Vermont Oxford Network (VON) data over an eleven year period, a twelve center study by Wallace et al., a sub-analysis of an RCT by Andrikopolou et al., and a single-center study by Townsel et al.^25,58–60^ An earlier study surveying infants born in Washington State between 1987 and 1995 did show a relative risk of 1.6 for RDS for Black infants compared to white infants.^61^

RDS-related mortality has declined significantly in the past two decades, with a particularly sharp decline following FDA approval of surfactant replacement therapy in 1990.^62,63^ However, the decline in RDS-mortality during this period was steeper for white infants compared to their Black counterparts. One population-based national study showed an increase in the relative risk of RDS-related mortality for Black infants between 1987 and 1995, compared to white infants.^63^ Other studies have reported similar worsening of the disparity between Black and white infant mortality related to RDS in this same time period surrounding the introduction of surfactants.^64–67^ A study of over 2 million infants born in Michigan between 1989 and 2005 also showed an increased risk of RDS-associated mortality for Black compared to non-Hispanic white infants.^68^ More recently, Karvonen et al. showed that Black infants with RDS were at increased risk of both readmission and death following neonatal discharge.^56^ Several theories have been proposed to explain the discrepancy between RDS rates and RDS-related mortality in Black infants, including a lack of response to surfactant in Black infants as a possible contributor, although differences would not be substantiated by a biologically plausible difference by the social construct of race.^65^ Other proposed mechanisms for lower rates of RDS in Black neonates have included a theory of accelerated lung maturation in Black neonates compared to their white counterparts secondary to environmental eposures.^69,70^ Black infants are more likely to experience stress in utero as they are more likely to be born in the setting of preeclampsia and placental insufficiency and more likely to be small for gestational age.^25,59, 61^ It is plausible that higher levels of stress exposure can lead to earlier surfactant production, and thereby explain the lower rates of RDS found in this population although this has not been proven.

### Bronchopulmonary Dysplasia and Pulmonary Hypertension

BPD is a major comorbidity of extreme preterm birth characterized by airway, alveolar, and vascular malformation and disruption of development and is distinct from acute RDS. Approximately 25% of infants with moderate to severe BPD develop pulmonary hypertension, a condition with a considerable mortality risk.^71–75^ Several studies have investigated racial and ethnic disparities in BPD and pulmonary hypertension with varied findings.

Early studies that sought to identify risk factors for BPD included race in their analyses and did not find it to be a significant risk factor.^76,77^ Using a tangential outcome, Palta et al. showed no difference in oxygen dependence at 30 days between non-Black and Black very low birth weight (VLBW, <1500 g) infants in an unadjusted model.^78^ After adjusting for several variables, non-Black infants were at elevated risk comparatively. ^78^ Several studies dedicated to investigating racial disparities were conducted thereafter. Petrova et al. detected no difference in incidence of BPD by race/ethnicity in a study of <32 week infants in 2003.^23^ Ryan et al. conducted a sub-analysis of the Prematurity and Respiratory Outcome Program (PROP) study including <29 week infants and found that Black infants had no difference in risk of BPD in an unadjusted model, and a lower risk of BPD compared to white infants when adjusted for GA although concerns have been raised about GA dating differences.^34,79^ Anderson et al. also found a reduced risk of BPD for Black infants compared to white infants who were born between 22–28wks GA, but no difference for those born between 28–36wks GA.^11^ In a population-based study investigating risk factors for a combined outcome of BPD and mortality, Lapcharoensap et al. described that Black birthing persons had a reduced risk for the combined outcome among <30 week GA infants.^80^

Fewer studies have concluded Black infants are at increased risk of BPD. Comparing conventional and fetus-at-risk approaches in statistical analysis, Janevic et al. described Black infants at a higher risk of BPD, with risk ratio of 1.64 (95%CI 1.38–1.95) in conventional model and risk ratio of 5.00 (95%CI 2.94–8.53) in their fetus-at-risk model.^24^

In regards to outcomes for infants with BPD after hospital discharge, despite finding a decreased risk of BPD for Black infants in the PROP study, Keller et al. also described Black infants at higher risk of post-prematurity respiratory disease, an outcome including respiratory medications, hospitalization, persistent respiratory symptoms, and home technology dependence.^79,81^ Similarly, in a population-based study in California, Karvonen et al. found that Black very preterm infants with BPD were more likely to be readmitted after hospital discharge.^56^

In the pulmonary hypertension literature, results are more uniform, with Black infants consistently at elevated risk for PPHN in the setting of BPD.^24,82,83^ In the pediatric population, significant racial variability in prevalence and subtype of pulmonary hypertension has been described that reflects neonatal trends. PPHN was most prevalent among Black infants, and Black children had a higher mortality risk driven by lung disease-associated pulmonary hypertension.^84^

### Necrotizing Enterocolitis

NEC is characterized by ischemic necrosis of the intestinal mucosa, and is the leading cause of gastrointestinal-related morbidity and mortality in neonatal intensive care units^.85–87^ Advancements in the understanding of NEC pathophysiology and risk factors have led to improvements in the overall incidence of NEC and NEC-associated mortality over time, although mortality remains high and racial disparities in NEC have been observed and persist.^85^

As early as 1991, Uauy and colleagues published that Black VLBW male infants had 2.3 times greater odds of NEC compared to non-Black infants.^88^ A decade later, Llanos et al. also described a higher incidence of NEC for Black infants compared to white infants.^89^ Building on this work, Guthrie et al. used an administrative database for births across 24 states between 1998–2000 among neonates between 23–34 weeks GA, and found that Black infants developed NEC more often than other infants.^90^ Several studies followed with similar findings that non-Hispanic Black infants were more likely to develop NEC and experienced higher rates of NEC-associated mortality, compared to infants of other races even after adjusting for birthweight and other clinical and demographic characteristics.^91–93^

When stratifying by GA, Anderson et al. found that Black infants born between 32–34 weeks (OR 1.49; 95%CI 1.03, 2.16) and 34–36 weeks (OR 2.29 95%CI 1.14, 4.58) were more likely to develop NEC than non-Black infants, however no difference was found in the <32 week GA stratification groups, suggesting that differential care practices for older infants may modulate the inherent risk based on immaturity.^11^ Janevic et al. found that with conventional modeling, Black infants had a borderline increased risk of NEC (ARR 1.34; 95%CI 1.09, 1.93), compared to white infants, whereas with the fetus-at-risk approach, Black infants had 4.4 (95%CI 2.98, 6.51) times the rate of NEC compared to white infants, suggesting that previous studies using conventional analyses may have underestimated the Black-white disparity in NEC.^24^

In regards to NEC-associated mortality, Seeman et al. found that the risk of NEC-mortality was 3.1 times higher (95%CI 2.8, 3.4) among Black infants compared to infants from all other race categories.^94^ Similarly, Jammeh et al. found that Black infants also had a higher rate of NEC-associated mortality (AOR 1.35; 95%CI 1.15, 1.58).^95^ Across studies, the general consensus is that despite adjustment for several sociodemographic and clinical factors, the Black-white disparities in NEC and NEC associated mortality have persisted over time.^55,58,85^

### Intraventricular Hemorrhage

IVH, resulting from a fragile and highly vascularized germinal matrix, is a common complication of extreme preterm birth, with reported rates of about 30–35% in infants born <28 weeks GA.^96–98^ Typically seen in the first week of life, the diagnosis of IVH places a neonate at increased risk of death, post-hemorrhagic hydrocephalus, and/or long-term adverse neurodevelopmental outcome, including developmental delay, cerebral palsy, blindness, and/or deafness.^99–101^ Though these are more commonly seen in infants with severe IVH (grades 3 or 4), milder grades of IVH have been increasingly associated with adverse neurodevelopmental outcomes as well.^102,103^ Given its many long-term complications, IVH can impact families for many years to decades after neonatal discharge.

Literature investigating Black-white disparities in IVH have yielded conflicting results. Qureshi et al. found that Black infants had a 2-fold increased risk of IVH and IVH-related mortality compared to white infants, while Shankaran et al., who had previously reported African ancestry as protective, reported a significantly increased risk of grade 2–4 IVH for this same group compared to white infants in their 2014 study.^104–106^ Similarly, Siddappa et al. showed that Black infants were disproportionately burdened by severe IVH compared to their representation in the study (80% vs 54%).^107^ Analysis of Vermont Oxford Network (VON) data over an 11 year period showed this same disparity, with Black infants displaying higher rates of severe IVH than white infants under 29 weeks GA.^58^ Wallace et al. also demonstrated similar findings, with increased risk of periventricular, intraventricular, and intracranial hemorrhage for Black infants compared to white infants.^59^ Despite several studies demonstrating increased risk of IVH for Black infants, several others have not shown this association.^11,23^ Townsel et al. initially found significantly higher rates of IVH for Black infants when compared to white infants (OR 2.01, p <.001), however this effect was no longer statistically significant after controlling for covariates including GA and exposure to antenatal steroids.^25^ Janevic et al. found no significant difference in risk of IVH in Black infants compared to white infants using a conventional analysis; however, when using the fetus-at-risk approach, they did find that Black infants had a 2.73 (95%CI, 1.63–4.57) times higher rate of IVH.^24^

Mortality after IVH may occur due to catastrophic hemorrhage leading to hypovolemic shock, secondary to other severe diseases such as sepsis or respiratory failure, or due to compassionate application of comfort, or palliative care. We did not find literature describing racial disparities in IVH-related mortality, but variation in IVH-related mortality by race could be mediated through differences in geographic, cultural, or center-specific palliative care patterns, which also renders IVH-associated mortality particularly vulnerable to variation based on race. Given concern for the disparities that exist in patterns of acquired brain injury described here, there is plausibility for ongoing disparities in longer term neurodevelopment, which are further discussed in this issue of Seminars in Perinatology by Fraiman et al.^148^

### Retinopathy of Prematurity

ROP is a neurovascular retinal disease marked by immature retinal vessels, which may cause retinal detachment resulting in functional or complete blindness in infants.^108^ Several studies have reported that Black infants experience a reduced risk of ROP compared to white infants.^11,35,37–41,59^ However, in a retrospective cohort with data from 1997–2005, Lad and colleagues found no racial differences comparing populations of infants with and without ROP, even after accounting for infants with significantly lower birth weight.^36^ Conversely, in crude logistic regression models, Townsel et al. found that Black infants had an increased odds of ROP (OR 2.51, 95%CI 1.97, 3.19).^25^ Similarly, using a fetus-at-risk approach and Cox-proportional hazards regression, Janevic et al. found that Black infants had 2.98 times the rate of ROP compared to white infants.^24^ Recently, a commentary on conflicting literature describing racial disparities in ROP with additional thoughtful considerations regarding social determinants of health and methodological limitations was published.^147^

### Patent Ductus Arteriosus

The PDA is a remnant of fetal circulation that can contribute to hemodynamic and respiratory morbidity when it fails to constrict after preterm birth. The degree of patency plays a role in whether the flow of blood through the PDA will impact cardiac function, or more often impact a preterm infant’s respiratory status through pulmonary overcirculation. There has been a suggested association between a persistent PDA and an infant’s risk of developing BPD, as well as contributing to other conditions comorbid with preterm birth such as NEC and pulmonary hemorrhage.^109–111^

While studies have investigated potential associations between many factors and a persistent PDA, a difference based on race or ethnicity has not been shown.^23^ The persistence of the PDA is so common amongst infants born preterm, and the severity difficult to ascertain in large administrative datasets that most studies examining racial differences in neonatal outcomes have not analyzed the presence of PDA stratified by race.^11,24,25,55,59^

However, the response to various treatment strategies to constrict or close the PDA in order to prevent its potentially deleterious impact on growth, cardiac, and respiratory function has been proposed to differ by race and ethnicity, as seen in several studies that link increased ductal constriction to non-white race while being treated with indomethacin.^112,113^ Waleh et al. investigated gene expression first in animal models, and then in human fetal tissue obtained in the second trimester, attributing the non-white response to indomethacin to two potential candidate genes involved in vasoconstriction and dilation, which attempted to establish a genetic basis to the clinical phenomenon of increased responsiveness to indomethacin therapy in non-white infants. The authors of the study on fetal tissue conclude that non-white infants have a genetic difference compared to white infants that may mediate a differential response to treatment for PDA. However, the study did not collect prospective data regarding self-identified race of those who donated the fetal tissue—over 75% of the samples were thought to reflect that of non-white patients. Additionally, the proposed genetic differences underlying response to PDA treatment could be confounded by unmeasured environmental exposures, such as neighborhood, poverty, education, pollution exposure, etc., that may explain the observed differences as they are not controlled for in these studies. Additional studies are needed to understand variable responsiveness to PDA treatment.

### Sepsis

Sepsis is a potentially devastating comorbidity of preterm birth, thought to be multifactorial, related to decreased immune protection, skin immaturity, lack of healthy microbiome development, as well as affected by care practices such as need for central lines and risk of hospital acquired infections.^114^ Early and late onset sepsis (EOS and LOS) are relatively rare, about 10–20 cases per 1000 live births for preterm infants, but mortality after sepsis for extremely preterm infants approaches 50%.^115,116^

While some studies have shown no difference by race/ethnicity for bacteriologically confirmed sepsis, Wallace et al. found a significantly increased risk for Black preterm infants to develop sepsis compared to white infants.^23,59,116^ Using the VON network database, Boghossian et al. found that although the discordant risk of sepsis in Black infants narrowed between 2006–2017, the risk of sepsis remained disproportionately high for Black infants compared to white infants.^58^ Three studies used a surveillance cohort of preterm and term infants over different years and across different sites with similar findings.^117–119^ Nanduri et al. and Weston et al. described a higher risk of early onset sepsis for Black infants compared to white infants during their study periods 2006–2018 and 2005–2008, respectively.^117,119^ Hamdan et al. and Nanduri et al. described a decreasing risk of early-onset Group B streptococcus (GBS) sepsis among Black infants, and a higher risk of late-onset GBS sepsis among Black infants.^117,118^

GBS is a major pathogen that has been targeted for universal screening and prophylactic treatment. Although universal screening has resulted in reduction in early onset GBS sepsis over time for all racial groups, concern remains for disparate colonization and screening and prophylactic treatment practices and a higher burden of GBS-disease among Black infants.^117,120–122^

## Mechanisms of disparities

### Structural and Institutional Racism Prior to Birth

Racism is recognized as a core determinant of health with consequences for the health of children and their families.^9^ In the U.S., structural racism manifests as systematically discriminatory laws and practices that have resulted in a disparate distribution of goods, services, and opportunities for Black communities.^123^ Structural racism has been directly associated with poor perinatal outcomes including preterm birth, infant mortality, small for gestational age status and postnatal outcomes including a combined outcome of mortality and severe preterm comorbidities, IVH, frequent acute care visits, rehospitalizations, and post-discharge mortality.^124–128^ Proposed pathophysiological mechanisms linking segregation and adverse outcomes include environmental toxicants such as air pollution and lead exposure, as well as chronic stress and allostatic load.^10,34,129–131^

### Patient Experience of Care

Qualitative studies elevating the voices of Black birthing people in the U.S. have validated concerns for disparate quality of care and additionally have exposed experiences of racial discrimination.^132,133^ Other studies have highlighted concerns Black families have had with nursing support, lack of compassionate and respectful communication, and less attentive care of their infants.^134^ Additionally, Sigurdson et al. described 324 accounts by healthcare providers and family advocates of disparate and privileged care by socioeconomic or racial group in a single center.^135^ Centering the margins by prioritizing the perspective of socially marginalized groups as the core of the discourse is a cornerstone of critical race theory (CRT), a methodology based in racial equity and social justice.^136^ Community partnerships can result in fresh perspectives and enhance relevancy of scientific work for investigations around racial disparities.^136^

### Peripartum Quality of Care

Quality of neonatal healthcare can vary across centers and regions; the extent of the variations and their contribution to racial disparities in outcomes has been an important area of investigation.^130^ Several studies have shown that Black infants are more likely to be born at hospitals with lower care quality scores and higher morbidity/mortality rates.^130,137,138^ Glazer et al. studied 40 hospitals in New York City, finding that Black infants were more likely to be born at centers with higher complication rates, with approximately 33% of Black infants born at hospitals in the highest morbidity and mortality tertile, compared to 10% of white infants.^138^ Howell et al. found similar results, with a 20% risk difference between Black and white very preterm infants of being born at a hospital in the highest tertile for morbidity and mortality.^137^ They further reported that when compared to other major determinants of neonatal outcomes such as infant health risk, maternal health risk, and SES, differences in hospital of birth accounted for 40% of the disparities in morbidity and mortality seen between Black and white infants.^137^ The authors suggest that despite known regional differences in overall quality of neonatal care, Black infants are more likely to be born in lower quality neonatal intensive care units (NICUs). This finding was replicated by Horbar et al., wherein the authors found that even after controlling for region, Black infants were more likely to be born in NICUs with lower Baby-MONITOR scores, a composite metric of NICU quality of care.^130^

### Postpartum Quality of Care

Neonatal transport is known to put preterm neonates at risk for complications compared to being inborn at a hospital able to provide the appropriate level of care intrapartum and immediately postpartum.^139,140^ Variation in the incidence, timing, and method of transport between hospitals by race and ethnicity has not been explored extensively but may be an area of future consideration as a mediator of racial disparities in preterm outcomes.

Within NICUs, racial and ethnic disparities in standards of care have been described across several standards of care including administration of antenatal steroids and breastmilk feeding, as well as composite measures of quality care metrics. ^26–31^ Standards of care including breastmilk feeding, GBS treatment, and antenatal steroids are important mediators in pathophysiological pathways that contribute to preterm comorbidities such as NEC, IVH, RDS, sepsis, and BPD.^120,141–143^ Equity focused quality improvement (EF-QI) has been proposed as a tool to reduce neonatal disparities, given that without an equity focus quality improvement interventions risk mitigating, perpetuating, or worsening disparities.^144^ EF-QI specifically prioritizes and addresses the needs of disadvantaged populations throughout a QI initiative from conception to analysis to dissemination.^144,145^ Overall, gaps in care practices that reduce the risk of preterm comorbidities, such as timely administration of antenatal steroids, is narrowing over time.^58^ Furthermore, diversifying the neonatal workforce may improve culturally competent care as racial concordance of providers and newborns has been associated with improved infant outcomes.^146^

## Looking to the future

Although at times the data for racial disparities in preterm comorbidities is conflicting and complicated by varying methodological approaches, it is clear that preterm birth and thus its resultant comorbidities disproportionately burden Black infants and their families. The differential risk of developing comorbidities for Black infants due to inequities in preterm birth is concerning, and thus focusing on reducing disparities in preterm birth is likely to have the largest population-level impact. Preterm birth and its complications have the potential to severely impact the lifespan and quality of life of infants. However, given the paucity of effective preterm birth prevention strategies, preventing the development of comorbid conditions of prematurity, combined with timely and effective treatment of conditions when they do arise, is necessary. Accomplishing these goals equitably will require dismantling structural racism and other societal systems of oppression.

## Supplementary Material

supplementary table

## Abbreviations:

CPAP: continuous positive airway pressure machines
RDS: respiratory distress syndrome
BPD: bronchopulmonary dysplasia
PPHN: persistent pulmonary hypertension
NEC: necrotizing enterocolitis
IVH: intraventricular hemorrhage
ROP: retinopathy of prematurity
PDA: patent ductus arteriosus
LBW: low birth weight
GA: gestational age
VON: Vermont Oxford Network
VLBW: very low birth weight
EOS: early onset sepsis
LOS: late onset sepsis
GBS: group B streptococcus
