# Supplementary material for: Racial disparities in the development of comorbid conditions after preterm birth: A narrative review
Source: Semin Perinatol. Author manuscript; Available in PMC 2025 Feb 19. (PMC11837808; doi:10.1016/j.semperi.2022.151657)
Supplement: supplementary table [file NIHMS2046575-supplement-supplementary_table.docx]

| Table 1. Study characteristics and summary of main findings included in narrative review, by comorbidity | | | | |  |  |
| --- | --- | --- | --- | --- | --- | --- |
| Comorbidity | Author (s), Year | Study design | Center type and database | Population Characteristics | Relevant Outcome | Summary of Relevant Findings |
| Respiratory Distress Syndrome |  |  |  |  |  |  |
|  | Anadkat, 2012 | Retrospective cohort | Multicenter database (Northern California Kaiser Permanente Medical Care Program | 34-42wk GA infants in 2000-2009 (n=286,454) | Respiratory distress syndrome (RDS) defined by respiratory distress on clinical exam and chest X-ray consistent with RDS (diffuse reticulogranular pattern with air bronchograms) | White race/ethnicity independently increased risk for RDS despite GA. Adjusted odds ratio of RDS for Black infants compared to white infants was 0.66 (95% CI 0.50-0.87) |
|  | Andrikopoulou, 2021 | Secondary analysis of RCT | Parent trial by National Institute of Child Health and Development Network of Maternal Fetal Medicine Units | 34-36wk GA infants (n=2331) | Severe respiratory morbidity (SRM) and RDS in infants exposed vs. not exposed to antenatal steroids. RDS not specifically defined. SRM was a composite outcome defined by CPAP or HFNC need for ≥12 hours, oxygen requirement of at least 0.3 for ≥24 hours, mechanical ventilation, ECMO, stillbirth and neonatal death <72hrs. | RDS lower in Black infants in the non-ANS (P=.01) but not in ANS group (P=.06). SRM lower in Black infants in ANS group (P=.01) but not in the non-ANS group (P=.06) |
|  | Hamvas, 1996 | Retrospective cohort | Missouri State Department Database of infants born to residents of St. Louis | Infant with birthweights 500-1500g, born in two time periods between 1987-1989 and 1991-1992 (n=1563) | Infant mortality | Bigger declines seen in white infant mortality rate after surfactant introduction than Black infant mortality rate. The relative risk of neonatal death for black infants went from 2.5 to 2.9 during from first to second period. Black VLBW infants less likley to receive surfactant than white infants (52% vs. 65%, p=.04) |
|  | Hessol, 2005 | Retrospective cohort | California linked birth-infant death data | Sinleton infants born 1995-1997 (n=1,277,393) | RDS cause-specific infant mortality. RDS as defined by ICD-9 code. | Infants born to Black birthing persons had significantly higher neonatal mortality (36.79 vs. 14.66 per 100,000 live births, p<.05)) and higher postneonatal mortality (11.93 vs. 0.98 per 100,000 live births, p<.05) due to RDS than those born to white birthing persons. |
|  | Malloy, 2000 | Population-based cohort | United States Vitals Statistics Tapes linked birth and death certificate files | Infants born 1987-1995 (n=23,958,870) | RDS cause-specific infant mortality. RDS as defined by ICD-9 code. | Mortality due to RDS decreased by 56% over 9-yr period around introduction of surfactant (84 to 37/100k life births).  Crude RR for RDS-related mortality for black infants compared to whites rose from 2.02 to 2.75 (p<.001) in that same period of time (due to sharper decline in IMR in white infants compared to Black infants) |
|  | Muhuri, 2004 | Retrospective cohort | National Center for Health Statistics Database | Singleton births between 1989 and 1991, and 1995-1995 (n=23,355,912) | RDS cause-specific infant mortality. RDS as defined by ICD-9 code. | Black infant RDS-related mortality significantly higher than white counterparts in both time periods (RR=2.86 from 1989-1991, RR=3.18 from 1995-1997) |
|  | Ranganathran, 2000 | Retrospective cohort | Linked birth/death certificate data for all US live births 1995, 1998 and 1991 | All liveborn infants 500-1499 (n=118,654) | RDS cause-specific infant morality. RDS as definited by ICD-9 code. | Rate of decline in mortality risks caused by RDS + all respiratory causes were greater for non-Hispanic white (NHW) infants than African American (AA) VLBW infants. OR of deaths by RDS declined by 34% in NHW and only 16% in AA between 1988 and 1991 (p <.01). For all resp causes: OR decrease by 41% in NHW and 22% in AA (P<.01). |
|  | Strandjord, 2000 | Retrospective cohort | Washington State Department of Health database | All infants born in Washington State between 1987-1995 whose birthing person's birth certificate data was available in the State database (n=46,246) | RDS rates and association of birthing person's low birthweight to RDS rates in infant. RDS defined by ICD-9 code in discharge summaries or birth certificate. | Relative risk of RDS 1.6 (95% CI 1.3-2.0) for Black infants compared to white infants. Low birthweight of birthing persons was associated with higher risk of RDS in both white, Native American and Black infants. |
| Bronchopulmonary Dysplasia | Lapcharoensap, 2015 | Retrospective cohort | California Perinatal Quality Care Collaborative database | VLBW infants born between 22-29 weeks GA, 2007-2011 (n=15,779) | Combined outcome of BPD or mortality prior to 36 weeks. BPD defined as continuous supplemental oxygen use at 36 weeks postmenstrual age. | Maternal Black race was associated with a reduced risk of combined outcome death or BPD (OR 0.79 95% CI 0.69-0.90) |
|  | Marshall, 1999 | Prospective observational cohort | North Carolina population database of 13 centers | 500-1500g infants born in 1994 (n=1244) | CLD defined as dependency on supplemental oxygen at 36 weeks postmenstrual age. | No difference in CLD by race in bivariate analysis of demographics |
|  | Palta, 1991 | Clinical trial | Newborn Lung Project, a multicenter study of 7 NICUs in Wisconsin and Iowa | <1501g preterm infants (n=581) | CLD defined as oxygen dependence at day 30 of life | No difference between Black and non-Black racial groups in oxygen dependence at 30 days of life (OR 1.1 95% CI 0.66-1.84). After adjusting for birthweight, GA, baseline severity score, apgar score, and gender non-Black race was associated with an increased risk of oxygen dependence in a joint multivariate model (OR 2.18 95% CI 1.00-4.74) |
|  | Rojas, 1995 | Prospective observational cohort | Single center | 500-1000g preterm infants born 1989-1991 who required mechanical ventilation (n=119) | Chronic lung disease defined as the need for supplemental oxygen for 28 days or longer during the first 2 months of life, in association with chest radiographic findings compatible with CLD (persistent hazy opacification or cystlike pattern of density and lucency) | Race was not a statistically significant predictor of CLD |
|  | Ryan, 2019 | Prospective observational cohort | Prematurity and Respiratory Outcome Program (PROP), a large multicenter prospective cohort | <29 week infants born in 2011-2013 (n=835) | BPD defined as supplemental oxygen requirement at 36 weeks PMA | The portion of infants with BPD was lower in Black infants (38% vs 45%) despite Black infants born at lower GA. Although in the unadjusted univariate logistic regression model the difference was not signficant (OR 0.77 95% CI 0.57-1.05). When adjusting for GA, intubation at birth, and antenatal steroids, the risk of BPD was lower for Black infants compared to white infants (aOR 0.60 95% CI, 0.42-0.85; P = .004) for Black infants compared with white infants after adjusting for GA. Despite the lower rate of BPD, Black infants had a higher rate of first-year post-prematurity respiratory disease (Black, 79%; white, 63%) |
|  | Keller, 2017 | Prospective observational study | Prematurity and Respiratory Outcome Program (PROP), a large multicenter prospective cohort | <29 week infants born in 2011-2013 (n=835) | Post-prematurity respiratory disease (PRD) defined as positive responses indicating respiratory morbidity on at least 2 caregiver questionnaires. Respiratory morbidity was defined as above: hospitalization for respiratory indication, home respiratory support, respiratory medication administration, and respiratory symptoms | Black infants were 1.59 times more likely to have post-prematurity respiratory disease (OR 1.59 95% CI 1.01-2.50) |
| Pulmonary Hypertension | Hernandez-Diaz, 2007 | Case-control surveillance study comparing infants with pulmonary HTN and healthy controls | Slone Epidemiology Center's Birth Defects Study, a case-control surveillance study that was designed to identify risk factors for birth defects in 4 metropolitan areas. | Infants >34 week GA born between 1998-2003 (n=642) | Pulmonary hypertension was documented by a ≥5% gradient between preductal and postductal oxygen saturation and/or by echocardiographic evidence (>95% of the cases had echocardiographic evidence) | Black infants were more likely to have a pulmonary hypertension compared to white infants (OR 3.0 95% CI 1.9-4.7). |
|  | Steurer, 2017 | Population-based retrospective cohort | California, using California Office of Statewide Health Planning and Development database | Infants >/=34 weeks GA born between 2007-2011 (n=1,781,156) | Persistent Pulmonary Hypertension (PPHN) defined by ICD-9 codes | Black infants were more likely to have a pulmonary hypertension compared to white infants (cOR 1.7 95% CI 1.4–2.0), which persisted after controlling for several other variables (aOR2.3 95% CI 1.4–3.7). |
| Intraventricular Hemorrhage |  |  |  |  |  |  |
|  | Ashajythi, 2019 | Retrospective cohort | Single-center, NICU at Hennepin County Medical Center, Minneapolis, MN | <29wks GA, 2008-2013 (n=101) | Predictors for severe IVH | African American race was associated with severe IVH (p=.031). AA infants made up 54% of study population and 80% of severe IVH infants and 50% of mild/no IVH. |
|  | Shankaran, 2014 | Case-Control Study | Multi-center, 24 Hospital US and Sweden | AGA infants, birthweight 500-1250g with exposure to at least 1 dose of antenatal steroids, and grade 2-4 IVH, born 2007-2012 (n=1111) | Differences in risk factors for IVH between races | White race was associated with decreased risk for IVH (OR 0.26, p .012). Among African American infants, having >1prenatal visit was associated with decreased risk of IVH (P=0.2) |
|  | Qureshi, 2013 | Retrospective cohort | NCHS Multiple Cause of Death data | All IVH-related mortalities reported between 2000-2009 (n=3249) | IVH, IVH-related mortality | Incidence rates of IVH were higher among AA (16/100k live births) than whites (7.8/100k live births). AA infants had 2-fold higher risk of IVH-related mortality compared with whites (RR 2.0). |
| Sepsis |  |  |  |  |  |  |
|  | Hamdan, 2021 | Population-based surveillance cohort | CDC Active Bacterial Core surveillance cohort of one states' data | Infants <90 days with GBS disease, 2009-2018 (n=356) | Invasive GBS disease was defined as isolation of S. agalactiae from a sterile site (eg, blood, cerebrospinal fluid [CSF], joint, muscle, bone, pleura, pericardium, perineum) using conventional microbiological methods. EOGBS was defined as invasive GBS disease in infants aged 0–6 days after birth and 7–89 days for LOGBS. | EOGBS rates have decreased significantly among Black infants and remained relatively stable among white infants, to similar rates at the end of the study period. LOGBS rates are signfiicantly higher for Black infants compared to white infants during study period, and declined significantly among Black infants yet stayed relatively stable in white infants. There was a decreased risk of GBS disease in white compared with Black infants (RR 0.43; 95% CI 0.33–0.54; P < .001). |
|  | Nanduri, 2019 | Population-based surveillance cohort | CDC Active Bacterial Core surveillance program of 10 states' data | Infants <90 days with GBS disease, 2006-2015 (n= 2,664) | Invasive case was defined as isolation of GBS from a normally sterile site, early onset (0-6 days), late onset (>7 days). | Statistically significant decline in EOGBS rates among both White infants (from 0.29 to 0.15 per 1000 live births; P < .001) and Black infants (from 0.76 to 0.55 per 1000 live births; P = .04), but EOGBS rates were 2.4 times higher in Black infants compared with White infants. Black infants had higher LOGBS compared to white infants (RR 2.9 95% CI 1.9-4.4) |
|  | Weston, 2011 | Population-based surveillance cohort | CDC Active Bacterial Core surveillance in 4 sites | 22-37 wks GA, 2005-2008 (n=658) | Early onset sepsis: one or more bacterial organisms isolated from either blood or cerebrospinal fluid (CSF) in the first 72 hours of life | Black preterm infants had the highest incidence (5.14 cases/1000 live births) and case fatality (24.4%) |
|  | Stoll, 2020 | Prospective cohort | 18 centers of the Eunice Kennedy Shriver National Institute of Child Health and Human Development Neonatal Research Network | Infants > 22 wks GA and 400g in 2015-2017 (n=9,575) | Early onset sepsis: isolation of a pathogen from blood or cerebrospinal fluid (CSF) culture obtained within 72 hours after birth and treatment with antibiotics for at least 5 days (<5 days if death occurred while receiving antibiotics) | No significant differences in EOS incidence were observed by sex, race, or ethnicity. |
| Necrotizing enterocolitis |  |  |  |  |  |  |
|  | Carter et al., 2008 | Descriptive secondary analysis using data from a larger longitudinal study | Preterm infants from a larger study in North Carolina, Ohio, Pennsylvania who were at high risk for NEC | VLBW infants (<1500 grams) or requiring mechanical ventilation at birth (n=134) | NEC was defined as either having confirmed documented NEC on radiograph by the hospital radiologist or a surgical intervention for NEC. Medical NEC was defined as a preterm infant who had pneumotosis intestinalis on x-ray and was treated with antibiotics for more than 2 days. Surgical NEC was defined as any surgical intervention (peritoneal drainage; exploratory laparotomy with diverting ostomy, intestinal resection or primary anastomosis or stoma creation) | Black infants had a significant correlation with NEC (beta: 0.18, p-value: 0.03) |
|  | Guthrie et al., 2003 | Retrospective cohort | 98 NICUs managed by Pediatrix Medical Group across 24 states, 1998-2000 | Infants born between 23-34 weeks GA (n=14,682) | NEC diagnosis was recorded in the database if a neonate had one or more of the following clinical signs: bilious, gastric aspirate or emesis, abdominal distention, occult or gross blood in stool without evidence of a rectal fissure; and had one or more radiographic findings (i.e., pneumatosis intestinalis, hepatobiliary gas, or pneumoperitoneum). | In univariate analyses, Black nenonates developed NEC more often than neonates of other race-groups (p-value <0.01). |
|  | Holman et al., 2006 | Retrospective cohort | National Kids' Inpatient Database for the year 2000 | Sample of 10% of the uncomplicated births and 80% of other paediatric discharges from short-term, non-Federal, general and specialty hospitals in 27 participating states within the US (n=4463) | NEC defined as hospitalisations with ICD-9-CM code for NEC. | NEC-associated hospitalisation rate among non-Hispanic Black neonates was 165.6 per 100, 000 live births [95%CI 137.2, 194.0], compared with 63.2 per 100,000 live births [95%CI 51.5, 74.9] for non-Hispanic white neonates. |
|  | Jammeh et al., 2018 | Retrospective cohort | Infants admitted in the first week after birth to neonatal intensive care units in the Pediatrix Medical Group from 1997 to 2015 | Infants ≤1500 g birth weight and ≤30 weeks gestational age (n=126,089) | Clinician diagnosed NEC | Non-Hispanic Black (aOR 1.31 95%CI 1.24-1.39, p-value <0.001) infants had a higher odds of developing NEC, compared with white infants. |
|  | Llanos et al., 2002 | Retrospective cohort | Six-county perinatal region in upstate New York | Live born infants during the 8-year study period (n=117,892) | Chart diagnosed NEC (modified Bell stage II and above) | The overall incidence of NEC for non-Hispanic black ifants was significantly greater than that for non- Hispanic whites (2.2 vs. 0.5 cases per 1000 live births, p-value = 0.00). |
|  | Seeman et al., 2016 | Retrospective matched case-control | United States Period Linked Birth/Infant Death data for 2010-2013 | Weighted NEC-deaths among 15 837 998 eligible live births (n=1,976) | NEC-associated infant mortality defined as presence of ICD-10 code as one of the causes of death on the death record. | The average annual NEC IMR was 28.6 deaths per 100 000 live births among among Black infants, compared with 9.3 per 100,000 among white infants. |
|  | Uauy et al., 1991 | Retrospective cohort | Infants admitted to centers participating in the National Institute of Child Health and Human Development (NICHD) Neonatal Research Network | VLBW infants (n=2,681) | NEC was defined prospectively and classified according to the modified Bell criteria as suspected (stages IA and IB) or proven NEC (stage IIA and more severe stages). | The odds of NEC was 2.3 times (95%CI 1.5, 3.4) higher for Black male infants, compared to non-Black male infants. |
| Retinopathy of prematurity |  |  |  |  |  |  |
|  | Chiang et al., 2004 | Population-based cohort | New York State, 1996-2000 | Newborn infants (n=15,691) | ROP diagnosis defined as presence of ICD-9-CM code on discharge | Black infants had a lower odds of ROP compared with infants of other race groups (OR 0.760 95%CI 0.685-0.843, p-value <0.001). |
|  | Lad et al., 2009 | Retrospective cohort | National Inpatient Sample from 1997 through 2005 | 34,000,000 live births | ROP identified by ICD-9 code | The authors found no racial differences in the incidence of ROP. |
|  | Port et al., 2015 | Retrospective cohort | Weill Cornell Medical Center and Columbia University Medical Center | Infants screened for ROP (n=1,354) | ROP and treatment-requiring ROP diagnoses were ascertained from a database of ROP screening examination results | Black infants had a lower odds of treatment-requiring ROP (OR 0.244 95%CI 0.095 -0.626). |
|  | Saunders et al., 1997 | Prospective cohort | Seventy neonatal intensive care units in 23 US participating centers in the Multicenter Trial of Cryotherapy for Retinopathy of Prematurity | Preterm infants with BW <1251g (n=4,099) | ROP was diagnosed with eye examinations between 28 and 49 days after birth | While ROP occurred with similar frequency in all racial subgroups, severe ROP was less common in black infants (p<0.001). |
|  | Tadesse et al., 2002 | Retrospective cohort | Georgetown University Hospital | ELBW infants (n=185) | ROP was defined according to the International Classification of ROP and infants were classified according to the highest stages of ROP observed in one or both eyes. Severe TOP was defined as stage III or greater | African-American infants had significantly lower odds of developing severe ROP (aOR 0.39 95% CI 0.16-0.97). |
|  | Yang et al., 2006 | Retrospective cohort | University Hospital of Cincinnati (1998-2003) | NICU-admitted premature infants weighing 401-1250 grams at birth (n=299) | ROP was diagnosed with information abstracted from chart review | Non-Black infants had a significantly higher odds of ROP warranting surgery (OR 4.23 95%CI 1.91-9.38, p-value <0.005) |
|  | Ying et al., 2015 | Observational cohort | Secondary analysis of data from the Telemedicine Approaches to Evaluating Acute-Phase Retinopathy of Prematurity Study | Infants with BW < 1251 grams (n=979) | ROP was diagnosed via serial diagnostic examinations in both eyes by ophthalmologists | White infants had a higher odds of refferal-warranted ROP (OR 2.76 95%CI 1.50-5.08) compared with Black infants. |
| Patent Ductus Arteriosus |  |  |  |  |  |  |
|  | Petrova et al., 2003 | Retrospective cohort | Single-center, level III NICU in New Jersey | 22-32 wk GA infants in 1998-2001 (n=1,006) | Incidence of PDA | No difference in rates of observed patency of the ductus arteriosus |
|  | Durrmeyer et al., 2010 | Propsective cohort | Single-center NICU in Germany | Preterm infants born < 28 weeks between 2003-2008 (n=111) | Infants with hemodynamically-significant PDA treated with ibuprofen, with genotyping for enzymes involved in ibuprofen metabolism | Enzyme polymorphisms were not associated with PDA response to ibuprofen, but ethnic group defined as "Caucasian" vs. "other" was associated with different response to ibuprofen in this study. |
|  | Waleh et al., 2015 | Laboratory based investigation | Fetal tissue collected and analyzed | 274 tissue samples of ductus arteriosus and aorta from mid-gestation pregnancies | Presence of candidate genes involved in iNO signalling and prostaglandin uptake | Non-white race was a significant predictor of infants who will close their PDA when treated with indomethacin |
| Multiple Comorbidities | Anderson, 2018 | Retrospective cohort | California Office of Statewide Health Planning and Development (OSHPD) birth cohort database | 24-36wks GA, 2007-2012 (n=245,242) | IVH, ROP, NEC, PVL defined by ICD-9 codes | No significant racial/ethnic differences in IVH and PVL in all GA groups, with the exception of slight increased OR 4.24 of IVH in Asian infants born 35-36wks GA. No Black-white disparities in BPD in 22-25wk GA, 32-34 wk GA, or 34-36 wk GA, but Black infants less likely to have BPD in 26-28 wk GA and 29-31wk GA (RR 0.70 95% CI 0.58 – 0.84 and RR 0.72 95% CI 0.53 – 0.98). No difference in NEC for 22-31wk GA, but increased risk for 32-34 wks and 34-36 wks GA (RR 1.49 95% CI 1.03 – 2.16,RR 2.29 95% CI 1.14 – 4.58). No difference in BPD for 29wks-36 wks GA, but decreased risk among Black infants for 22-25 wks and 26-28wks GA (RR 0.46 95% CI 0.37 – 0.63, RR 0.47 95% CI 0.28 – 0.80). |
|  | Boghossian, 2019 | Retrospective cohort | VON centers | 22-29wks GA, 2006-2017 (n=226,283) | RDS: room air Pao2 <50 mm Hg, room air with central cyanosis, supplemental oxygen to maintain Pao2 >50 mm Hg, or supplemental oxygen to maintain a pulse oximeter saturation >85% and a chest radiograph consistent with RDS within the first 24 hours of life. NEC: At time of surgery or postmortem or required ≥1 clinical sign (eg, bilious gastric aspirate, abdominal distension, occult blood in stool) and ≥1 radiographic finding (eg, pneumatosis intestinalis, hepatobiliary gas, or pneumoperitoneum). Early-onset sepsis: bacterial pathogen recovered from blood or cerebrospinal fluid on or before day of life 3. Late-onset sepsis: bacterial pathogen or coagulase-negative Staphylococcus recovered from blood or cerebrospinal fluid or fungus recovered from blood culture after day 3 of life. Coagulase-negative Staphylococcus infection also required ≥1 sign of generalized infection and treatment with ≥5 days of intravenous antibiotics. Severe IVH: grades 3 or 4 using Papile’s classification within 28 days of birth. Severe ROP: stages 3 to 5 based on a retinal examination before hospital discharge. CLD: any supplemental oxygen use at 36 weeks’ postmenstrual age or on oxygen at discharge at 34 to 35 weeks if transferred or discharged <36 weeks’ gestation | For Black versus white infants, RDS, EOS, sIVH, sROP, CLD, pneumothorax, and morbidity-free survival revealed a constant rate difference over time. EOS rates revealed occasionally slightly higher rates for African American infants, whereas sIVH was higher among African American than white infants for most of the years. RDS and sROP were lower among African American infants for some but not all years. CLD and pneumothorax were lower among Black infants throughout the study period. Morbidity-free survival was higher among African American infants for all years except one. The rate difference for NEC, and LOS remained higher for Black infants throughout the study period. However, the rates for these outcomes decreased faster over time for African American than white infants with a significant year-race interaction (LRT P < .0001, mortality; P < .0001, hypothermia; P = .028, NEC; P < .0001, LOS). |
|  | Janevich, 2018 | Population-based retrospective cohort | NY Statewide linked birth certificate data (data from Statewide Planning and Research Cooperative System [SPARCS]) | 24-31 wks GA, 2010-2014 (n=582,297) | BPD, IVH, ROP, and NEC were defined by ICD-9 codes | Conventional approach: Black compared with white infants had an increased risk of only bronchopulmonary dysplasia (adjusted risk ratio [aRR] 1.34 95% CI, 1.09-1.64) and a borderline increased risk of necrotizing enterocolitis (aRR 1.39 95% CI 1.00-1.93). In the fetuses-at-risk analysis, black infants had a 4.40 times higher rate of necrotizing enterocolitis (95% CI 2.98-6.51), a 2.73 times higher rate of intraventricular hemorrhage (95% CI 1.63-4.57), a 4.43 times higher rate of bronchopulmonary dysplasia (95% CI 2.88-6.81), and a 2.98 times higher rate of retinopathy of prematurity (95% CI 2.01-4.40). |
|  | Karvonen, 2021 | Retrospective cohort | California Office of Statewide Health Planning and Development (OSHPD) birth cohort database | <37 weeks GA, 2007-2011 (n=189,306) | Readmission and mortality among infants with BPD and RDS defined by ICD-9 codes | Black preterm (<32 weeks GA) infants with BPD were more likely to be readmitted (RR 1.3 95% CI 1.1-1.6 p<0.05); Black preterm infants (32-36 weeks GA) with RDS more likely to be readmitted than their white counterparts (RR 1.4, CI 1.2-1.6 p<0.05). Black preterm infants (32-36 weeks GA) with RDS more likely to die after discharge (RR 5.7, CI 1.8-12.2). |
|  | Ong, 2019 | Retrospective cohort | PPHNet registry: a multicenter registry enrolling at eight pediatric PH clinical programs in North America | <18 year olds enrolled in 8 PPHNet centers between 2014-2018 (n=1,417) | PPHN and BPD as defined by PPHNet registry | Among neonates, persistent pulmonary hypertension of the newborn (OR 4.07 95% CI 1.54-10.0; P = .0029) and bronchopulmonary dysplasia (OR 8.11 95% CI 3.28-19.8; P < .0001) were more prevalent among Black infants |
|  | Petrova, 2003 | Retrospective cohort | Single-center, level III NICU in New Jersey | 22-32 wk GA infants in 1998-2001 (n=1,006) | BPD was defined as a requirement for oxygen supplementation at 36 weeks postmenstrual age. IVH, PVL | The variation in the major neonatal morbidity such as BPD did not reach statistical significance when analyzed by race/ethnicity. No statistical difference seen rates of IVH and PVL by race in each gestational age category. |
|  | Townsel, 2018 | Retrospective cohort | Single-center in Connecticut | <37 weeks GA,1994 to 2009 (n=4,953) | RDS: clinical findings of respiratory distress and chest X-ray findings of diffuse reticulogranular pattern with air bronchograms. IHV: Papile classification of cranial ultrasound findings of blood in germinal matrix or ventricular system with or without ventricular dilatation. NEC: Bell’s classification (stage II and above). ROP: pediatric ophthalmologic exam using the international classification of ROP | There was a significant difference in RDS among Black neonates (BNs) (aOR 0.57, 95% CI 0.45-0.73; p < 0.001), but not after restricting to <28 week GA. Black infants were more likely to experience IVH (OR2.01 95% CI 1.48-2.70, p<0.001) compared to white infants. Black infants were more likely to experience NEC (OR 2.75 95% CI 1.64-4.46). Black infants were more likely to have ROP (OR 2.51 95% CI 1.97-3.19). There was no difference in IVH, RDS, ROP or NEC across REGs in very preterm (<28 wks GA) infants. |
|  | Wallace, 2017 | Retrospective cohort study | National, using data from the Consortium on Safe Labor that includes 12 centers | <37 weeks GA, 2002-2008 (n=19,325) | IVH, ROP, NEC, sepsis defined by ICD-9 codes | Sepsis, periventricular/intraventricular hemorrhage, ICH, and ROP significantly different by race (p</=0.01). When adjusting for study site, Black infants were significantly more likely to experience sepsis (RR 1.41 95% CI 1.27-1.57), peri- or intraventricular hemorrhage (RR 1.32 95% CI 1.04-1.69), intracranial hemorrhage (RR 1.52 95% CI 1.11-2.11), and retinopathy of prematurity (RR 1.41 95% CI 1.06-1.89). When adjusting for study site, there was no significant difference between Black and White infants for RDS (RR 0.99 95% CI 0.91-1.08) or NEC (RR 1.28 95% CI 0.82-1.98). |
